# Supplementary figures and images for: Identification and Functional Validation of the PeDHN Gene Family in Moso Bamboo
Source: Plants (Basel). 2025 May 19;14(10):1520. doi: 10.3390/plants14101520 (PMC12115333; doi:10.3390/plants14101520)

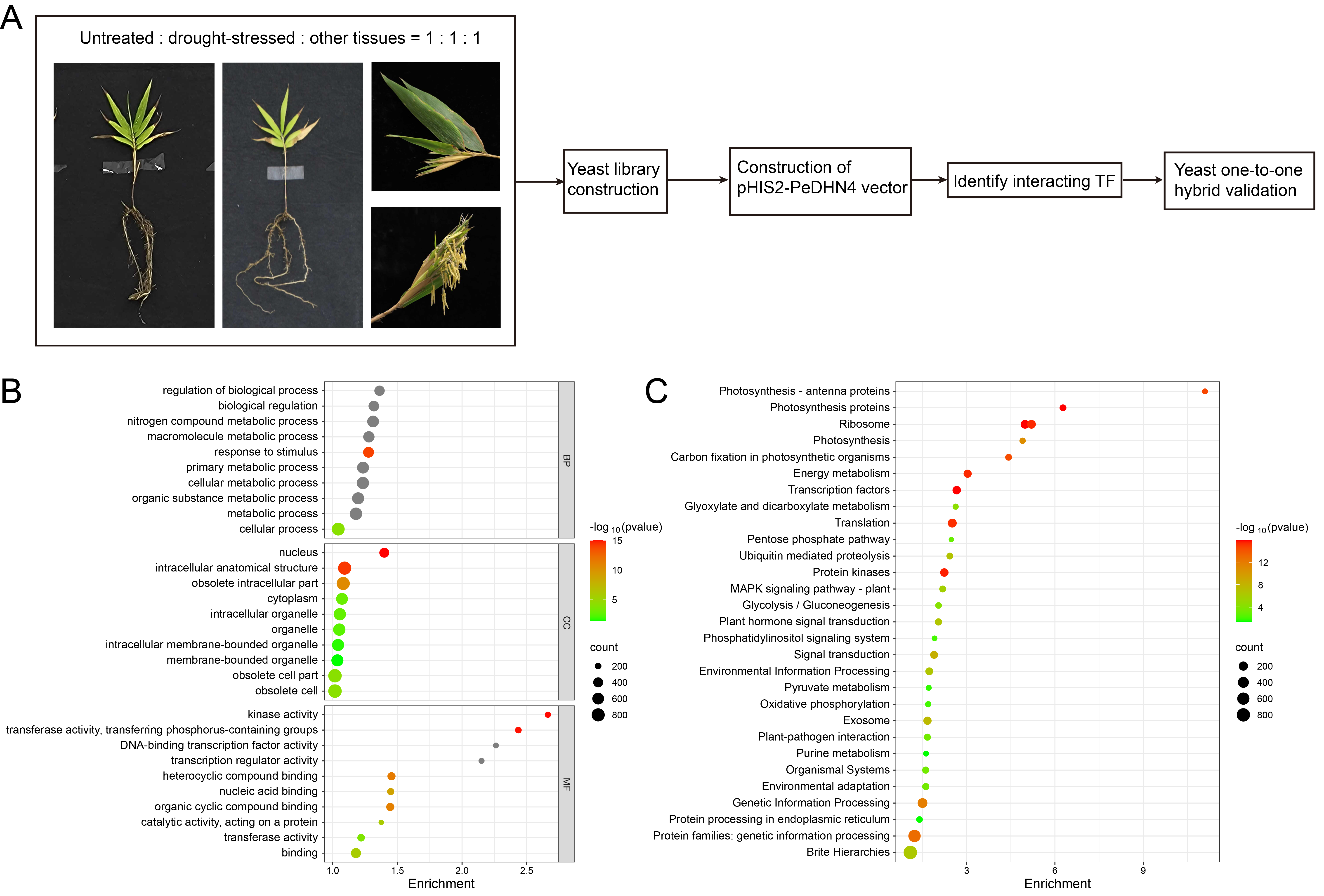

Supplement: Supplementary file 1 [file plants-14-01520-s001.zip › FigureS/Figure S2. Yeast One-Hybrid Library Construction Workflow and GOKEGG Enrichment Bubble Plots..tif]
